# Evaluating Public Health Interventions: A Neglected Area in Health Technology Assessment

**DOI:** 10.3389/fpubh.2020.00106

**Published:** 2020-04-22

**Authors:** Jovana Stojanovic, Markus Wübbeler, Sebastian Geis, Eva Reviriego, Iñaki Gutiérrez-Ibarluzea, Irene Lenoir-Wijnkoop

**Affiliations:** ^1^Department of Health, Kinesiology, and Applied Physiology, Concordia University, Montreal, QC, Canada; ^2^Montreal Behavioural Medicine Centre, CIUSSS du Nord-De-L'Île-De-Montréal, Montreal, QC, Canada; ^3^Department of Nursing Science, Hochschule für Gesundheit—University of Applied Sciences, Bochum, Germany; ^4^Osteba, Basque Office for Technology Assessment, Basque Foundation for Health Innovation and Research (BIOEF), Bilbao, Spain; ^5^Basque Foundation for Health Innovation and Research (BIOEF), Bilbao, Spain; ^6^Department of Pharmaceutical Sciences, Utrecht University, Utrecht, Netherlands

**Keywords:** public health, intervention, technology, HTA, survey, lifestyle and behavior, at-risk populations

## Abstract

**Introduction:** Public health (PH) interventions are crucial for ensuring sustainable healthcare services. Nevertheless, they represent a neglected area in the field of health technology assessment (HTA) due to various methodological issues and their complex design that goes beyond clinical setting. The present study provides an environmental scan of HTA initiatives related to the assessment of PH technologies on a global level.

**Methods:** We conducted a cross-sectional survey among 85 HTA-related European and international societies, health bodies, and networks from September 2018 to January 2019. The questionnaire contained four sections and 18 questions regarding activities related to the evaluation of PH technologies, information on existing PH technologies, and methodologies of assessment as well as barriers and facilitators to reaching a decision and implementing a PH technology.

**Results:** Among 52 survey responses, the majority of the respondents came from European countries (35%), followed by North American (27%), and South American (19%) countries. The main type of organizations covered by our survey included HTA agencies, public administrations, and research institutes. Seventy-one % of the institutions reported engagement in any aspect of HTA in the area of PH (*N* = 37). Among those, 81% evaluated less than 5 PH technologies from 2013 to 2018. The most common barriers for reaching a decision on PH technologies were lack of data, conflicting stakeholder priorities, and methodological issues. A total of 76 PH interventions were reported, and most cited initiatives were related to chronic disease screening, prevention of infectious diseases, and maternal, prenatal, and neonatal screening.

**Conclusion:** Our survey reported a rather limited involvement of HTA in the evaluation of PH technologies. In particular, an evaluation of behavioral and lifestyle interventions remains extremely rare. The implementation of collaborative HTA approaches in the setting of PH practice and policy needs to be prioritized and further strengthened. Moreover, ensuring reliable data structures and consolidation of HTA methods for the evaluation of PH technologies will be crucial for tackling the enormous burden of non-communicable diseases in societies.

## Introduction

Non-communicable diseases are the biggest drivers of global mortality, contributing to over 70% of total deaths in 2017. The vast majority of premature deaths occur in low- and middle-income countries in part due to behavioral risk factors such as increasing rates of tobacco and alcohol consumption, unbalanced diets, and physical inactivity (1).

Parallel to these devastating figures, the world population health is facing numerous additional threats including outbreaks of vaccine-preventable diseases, increasing antimicrobial resistance, population aging, as well as air pollution and climate change (2–4).

The sustainability of healthcare systems may be supported by investing more in health promotion, disease prevention, and early diagnosis rather than in disease treatment (5). With the most recent technological advancements as well as citizen empowerment, there is a tendency to move away from the “one size fits all” approach and to develop personalized approaches that would place citizens at the core of health systems (6).

Public health (PH) practitioners and institutions have a crucial role in tackling these challenges by bringing together health professionals, service providers, policymaking authorities, and governmental agencies to strive for healthy communities while ensuring social justice and equity.

Health technology assessment (HTA), as a multidisciplinary process, applies systematic procedures when evaluating health technologies in order to support and guide health policy decision making. HTA comprehensively appraises health technologies, focusing both on their direct effects, and indirect consequences (7).

Traditionally, the vast majority of HTAs have been concentrating on the clinical area, as well as on pharmaceuticals and/or medical devices as the main target. Lavis et al. provided an inventory of HTAs across nine diverse HTA organizations, conducted from 2003 to 2006, highlighting that only 5% of health technologies were PH interventions (8).

A systematic evaluation of effectiveness can be easily applied in the field of clinical interventions, while the complexity of PH interventions in the real-world setting still poses methodological challenges (9–11). A recent systematic review about HTAs of PH interventions reported a limited number of guidelines in this field. The authors also observed numerous, and often unjustified, changes to recommendations such as adaptations in the processes of quality evaluation, assessment of applicability, and integration of qualitative and quantitative evidence (12, 13). These methodological tweaks were mainly linked to the fact that complex PH interventions implement diverse methodologies that often fall outside a controlled experimental setup such as those processed in randomized controlled trials. However, this could not be a justification for the scarce number of PH technologies assessed by HTA bodies.

We performed a cross-sectional survey among international organizations, networks, and societies involved in HTA in order to: (1) analyze the magnitude of their involvement in the evaluation of PH technologies, (2) provide specific information on existing PH technologies as well as methodologies of assessment, and (3) understand barriers to assessing/reaching a decision on and implementing a PH intervention. For the purpose of this survey, PH was defined as all organized measures (whether public or private) to prevent disease, promote health, and prolong life among the population as a whole, involving a collaborative effort by all parts of the health sector working to ensure the well-being of society through comprehensive prevention, treatment, care, and support (14).

## Materials and Methods

### Survey Development

Three authors (JS, ILW, and IGI), executive members of the HTAi Interest Group Initiative for Public Health Outcomes Research and Measurement (INPHORM), developed a draft questionnaire in July 2018 following a series of discussion-based meetings and subsequent exchanges until consensus was reached. The draft survey was sent for additional consultation to the HTAi members present at the INPHORM business meeting during the HTAi Annual Meeting in Vancouver, Canada.

The final survey consisted of 18 questions across four different sections, including contact information, activities related to evaluation of PH technologies, PH intervention candidates, and questions about the process of reaching a decision on a PH technology (Supplementary Table 1). Briefly, the survey collected data on what kind of PH technologies and/or interventions had been assessed in the last 5 years as well as specific details of the technology/intervention. Moreover, the questionnaire encompassed a mapping of methodologies used in the assessment and of enablers and barriers to assessing, adopting, or delisting the intervention.

### Survey Distribution and Data Collection

#### Survey Software

The survey was implemented using an online survey management system (15) that ensured the confidentiality of the answers. An email explaining the purpose of the questionnaire and indicating the deadline for submitting responses was sent with the link to each of the participants,. The survey was distributed among the member lists of diverse HTAi partners: international societies, health bodies, and networks, including Health Technology Assessment international (HTAi), The International Information Network on new or emerging, appropriate use and re-assessment needed Health Technologies (EuroScan International Network e.V.), the European network for Health Technology Assessment (EUnetHTA), the International Network of Agencies for Health Technology Assessment (INAHTA), the National Health Service of the United Kingdom (NHS-UK), the York Health Economics Consortium (YHEC), the Institute for Quality and Efficiency in HealthCare (IQWiG), the Haute Autorité de Santé (HAS), the National Institute for Public Health and the Environment (RIVM), the European Public Health Association (EUPHA-HTA section), the Red Española de Agencias de Evaluación de Tecnologías Sanitarias (REDETS), the Red de Evaluación de Tecnologia en Salud de las Américas (RedETSA), and the Health Technology Assessment Asia Network (HTAsialink). It was sent out to a total of 85 recipients and was open from September 2018 to January 2019. Data extraction of the responses to the questionnaire was conducted by one author (ERR) and was cross-checked for consistency by two additional authors (JS and ILW). Any emerging discrepancies were resolved through discussion.

### Statistical Analysis

Descriptive statistics were calculated to summarize the sample. The statistical analysis was conducted in IBM SPSS Statistics 25 software and STATA version 12 software.

### ICHI Encoding

In addition to the statistical analysis, we tested the feasibility of using the International Classification of Health Interventions (ICHI) for categorization of the survey outcomes. The ICHI is an open-access classification available *via* an online database hosted by DIMDI (16). The ICHI is part of the WHO-FIC reference classifications, with the aim to develop a common tool for reporting and analyzing health interventions for statistical, quality, and reimbursement purposes. Given the paramount importance of PH within all health structures worldwide, PH represents an integrated component of the ICHI.

The coding for the present study was performed through searching for the intervention titles by a single pair of authors (MW and SG). If the feasibility testing proved to be positive, we would conduct a follow-up project by involving additional reviewers and ensuring the independent coding process (work in progress).

## Results

### Description of the Respondents

In total, 52 respondents from all continents answered the survey. The majority came from European countries (34%), followed by North American (26.9%) and South American countries (19.2%). Equal numbers of HTA agencies, public administration institutions, and scientific research institutes responded to the survey (*N* = 11 each). The remaining respondents included nine academic, six industry, and four hospital institutions. These results are reported in Supplementary Figures 1, 2.

### Public Health Intervention Candidates

Our survey yielded a total of 76 PH technologies reported by the respondent institutions. A wide variety of interventions was identified, targeting different populations and diseases. In general, the reported technologies could be roughly subdivided in the following groups: primary prevention (42.1%), secondary prevention (48.7%), tertiary prevention (5.3%), and others (policy, mixed, *etc*.) (3.9%). When sorting the answers according to health concerns, screening of chronic diseases showed the highest proportion of interventions (25%), followed by infectious diseases prevention, in particular vaccines and disease screening (21.1%), and maternal, prenatal, and neonatal screening initiatives (9.2%). The reported assessment of behavioral interventions was low both in the adult population (3.95%) as well as in children and adolescents (3.95%). Similar outcomes were found for environmental interventions such as tobacco cessation (3.9%) and mental health screening (3.9%).

The survey question on the most important reasons for conducting a PH assessment allowed several answers per respondent. The reason most frequently reported in this survey was the need to identify whether a PH intervention represents a better alternative to standard procedures (75%). Other important drivers were the need to evaluate the impact on change in healthcare practices (51.3%) and the impact of an intervention on healthcare budget/resources (48.7%) (data not shown).

### Assessment of PH Technologies

Out of the 52 respondents to the survey, 37 institutions reported engaging in any aspect of HTA in the PH area (71%). Thirty-four of these organizations were involved in assessment activities, including reviewing and/or generating evidence of effectiveness or cost-effectiveness (Table 1). The majority of respondents stated that their institution is involved in assessing the medical aspects of the technology (*N* = 32), followed by economic considerations (*N* = 25), organizational impact (*N* = 23), and societal consequences (*N* = 23) (Table 2).

**Table 1:** An overview of activities of 37 organizations engaged in PH technology assessment on (cost-)effectiveness (multiple-choice question).

**Categories**	***N***	%^*^
Assessment	34	92
Selection of topic/target (sub)populations	16	43
Identification of topic/of target (sub)populations	15	41
Dissemination and diffusion	14	38
Project prioritization	13	35
Implementation	13	35
Horizon scanning	12	32
Monitoring	12	32
Decisions	11	30
Design of the intervention/educational campaign/any form of care support	11	30
Coordination of activities	9	24
Other	3	8
Total number of answers	163	

* *Percentage of institutions that answered positively*.

**Table 2:** Answers regarding aspects of the PH technology assessment reported by 34 organizations involved in the assessment activities (multiple-choice question).

**Categories**	***N***	%^*^
Medical aspects	32	94
Economic evaluations	25	74
Organizational impact	23	68
Societal perspectives and consequences	23	68
Ethical considerations	16	47
Legal consequences	8	24
Other	4	12
Total number of answers	131	

*N, number of responses*.

* *Percentage of institutions that answered positively*.

Concerning the volume of evaluated PH technologies, 81% of the 37 institutions reported evaluating <5 PH technologies, while 27% of the institutions had evaluated only one PH technology since 2013. Various methodologies for assessing PH technologies were reported, the most common of which include HTA, budget-impact analyses, and multi-criteria decision analyses (Table 3).

**Table 3:** Methods, frameworks, or tools reported by 37 organizations engaged in PH technology assessment (multiple-choice question).

**Categories**	***N***	%^*^
Health technology assessment	32	91
Budget impact analyses	15	43
Multi-criteria decision analysis	7	20
EUnetHTA core model	5	14
Health impact assessment	4	11
Health technology reassessment	3	9
INTEGRATE-HTA model	2	6
Based on one of the four existing guidance documents	2	6
Program budgeting marginal analysis	1	3
Guideline for not funding health technologies (GuNFT)	1	3
Model for sustainability in healthcare by allocating resources effectively (SHARE)	0	0
Other	9	26
Total number of answers	81	

* *Percentage of institutions that answered positively*.

To the question “is your organization currently involved in or planning to launch an evaluation of a PH technology?,” 42% of the 52 survey respondents replied positively.

### Barriers to Reaching a Decision About Implementing a PH Technology

The most commonly reported barriers in reaching a decision on the adoption or delisting of a PH technology were lack of relevant data to conduct an assessment (54%), conflicting priorities among diverse stakeholders (43%), common methodological issues and lack of clear methodological frameworks to properly assess PH interventions through an HTA approach (32%), difficulty to assess the impact and to reallocate resources across and between programs or sectors of complex PH interventions (30%), and political challenges (30%) (Supplementary Table 2).

In regard to the barriers of implementation, 41% of the institutions reported not being engaged in decision implementation processes. Among the 22 institutions, the most common barriers involved in implementing decisions were clinicians' reluctance to change habits in their daily practice (24%), lack of staff and resources necessary to implement the intervention (22%), lack of funding for implementation (22%), perception that management priority is costing money while return on investment is not warranted (22%), and lack of perceived benefit related to the complex context of the technology/bundle of technologies (19%) (Supplementary Table 3).

### Coding According to the ICHI

In many cases it was necessary to describe the intervention with appropriate keywords since the exact title was not defined in the ICHI. A certain training period was required because of the structure of the ICHI codes; technical handling of the platform is very workable after a short orientation period.

A precisely descriptive ICHI code could rarely be assigned to the executed intervention. Often codes had to be used which would approximate the interventions. In addition, there were cases in which interventions corresponded to different codes regarding “target” and “action”; therefore, a combination of these codes would have been useful.

Furthermore, the PH interventions were not always described precisely, which meant several codes could be considered. This notwithstanding, after encoding for each of the reported PH interventions, it appeared that several answers could be clustered in a relatively small number of categories of the ICHI (Table 4).

**Table 4:** Answers coded with WHO International Classification of Health Interventions (ICHI) after a single-pair categorization (most mentioned categories: *N* > 3).

**Coding**	**Descriptors**	***N***
VFX.PP.ZZ	Counseling about other health-related behaviors	16
UA1.WG.QF	Economic incentives concerning products and technology in relation to health	15
DTB.DB.AE	Percutaneous administration of immunological agent	11
UA1.VC.ZZ	Public health surveillance concerning products and technology	11
UA1.VC.ZZ	Assessment of urogenital system, not elsewhere classified	10
NMF.AH.AC	Cervical papanicolaou smear	9
VFX.VC.ZZ	Public health surveillance concerning other health-related behaviors	6
UA1.AA.ZZ	Descriptor: assessment of products and technology	6
LCA.BA.BA	Mammography	5
DTB.AA.ZZ	Assessment of immunological system functions	4
VAB.RD.ZZ	Provision of products to support improved health behaviors relating to tobacco use	4
VAB.RD.ZZ	Provision of peer support for tobacco use behaviors	4
VAB.PM.ZZ	Education to influence tobacco use behaviors	4
VAB.PN.ZZ	Advising about tobacco use behaviors	4
UAC.AA.ZZ	Assessment of medication	4

## Discussion

The present survey involved 52 international HTA bodies and aimed to capture their engagement in the PH field as well as specific PH technology characteristics and methodologies of assessment. Around 70% of the responding institutions reported being engaged in PH assessments, mainly HTA agencies, research institutes, and public administration bodies.

The outcomes of this survey indicate that the assessment of PH interventions represents only a limited proportion of evaluations carried out by institutions engaged in HTA. Indeed over 80% of the institutions reported performing <5 PH technology evaluations since 2013.

Considering the PH intervention candidates, our survey reports a total of 76 PH technologies that had been evaluated by the institutions. Although the majority of the reported assessments focuses on primary and secondary prevention programs, it appears that in many cases the targeted populations are already benefiting from some form of clinical monitoring or care provisions, indicating that the prevention intervention approaches are more frequently delivered within the healthcare setting, thus not reaching the far higher number of concerned (at-risk) citizens who do not access healthcare services. Nevertheless, there is an increasing number of implemented initiatives that are linking primary care with sources of community support, but the evidence about their effectiveness and methodological rigor is limited (17). The key issue with building the evidence base for initiatives such as social prescribing as well as PH interventions in general lies in the fact that applying the golden standard of evidence (i.e., double-blinded randomized controlled trials) might not be adapted. Pragmatic trials, as a more naturalistic approach, might provide a better picture of effectiveness, while evidence summaries may include a realist synthesis as opposed to standard systematic review approaches (12, 18–20). This being said, it should be kept in mind that good evidence does not necessarily lead to good policy (21).

In our survey, <5% of the interventions focused on behavior. The evaluation of behavioral and lifestyle interventions remains extremely rare in spite of their importance for developing effective population health strategies and establishing health systems. This notwithstanding, PH researchers and other non-HTA professionals frequently study a variety of approaches for prevention interventions that directly target the general population (22, 23). However, associated recommendations and more generalized implementation of campaigns and interventions do not belong to their mainstream responsibilities. Moreover, very few behavioral trials have been registered and many of them lack transparent reporting (24). It is thus important to emphasize that rigorous conduct as well as transparent reporting need to be implemented.

The inadequacy of HTA from an overall PH perspective is also reported in literature (12, 25–27). One of the underlying reasons probably lays in the multi-dimensional character of PH, a field which is increasingly affected by a full range of extra-clinical circumstances (28, 29), including demographic transitions (30) and modifiable risk factors of disease development associated with unhealthy lifestyle habits in the general population (31–35). Lifestyle research has to do with socio-economic aspects including behavior sciences and drivers of individual choices; effective PH management therefore requires an integrated perspective on the health–disease continuum (36–40). The resulting uncertainties (41), the fact that benefits at the population level might not necessarily be synonymous to benefits for single individuals, as well as a lack of insight in the variability of the many underlying causal pathways (42) often lead to a de-prioritization of preventive interventions on the population level, emphasizing that collaborative approaches among different professionals (from PH practitioners to behavioral scientists, psychologists, community and social workers, *etc*.) will be crucial for implementing efficient and effective interventions once the decision has been made.

Not surprisingly, a median return on investment of PH interventions is rather high. A recent systematic review estimated that each pound invested in PH will lead to 14 pounds being returned to the health and social care economy. Yet again, many of PH interventions, in particular those associated with behavioral risk factors, do not get funded. One of the plausible explanations is that political incentives are lacking since the benefits of PH interventions usually arrive after the mandate of current politicians and policy makers (43). Furthermore, it could be related to a lack of interest due to the inexistence of promoters as it is the case of other technologies that are designed and marketed by companies with an obvious business interest in their marketing.

This might also explain—at least partly—the barriers for reaching a decision about a PH technology or the implementation of a PH intervention as mentioned by the respondents of our survey. Similar elements are reported in the recently published INAHTA viewpoint (44).

The distribution of our survey *via* the HTAi partners may have induced an underrepresentation of PH specialists and represent a limitation. Indeed, as mentioned above, HTA approaches are getting increasingly used by PH scientists as a recognized instrument for obtaining insight on various kinds of PH measures and their impact on health logistics/systems. Recent reports show that their studies are frequently supported by HTA organizations (45–48); this type of collaborations was not necessarily captured in the survey.

### ICHI Content Suitability

Basically, the suitability of the ICHI could be determined and we were able to code the PH interventions. We found multiple ICHI codes matching the survey data, which was also related to the inductive approach of our survey. To reach a certain level of specificity, the ICHI classification could be used to design future surveys that integrate codes and definitions to questionnaires. However, this will likely lead to an extension of the survey and fewer responses of individuals. There were clear differences concerning the accuracy of fit. Nevertheless, there has been coding in all four categories of interventions. Differences were found in the three axes of each ICHI measure (target, action, and means). In many cases, the means were coded as “other and unspecified means.”

Representing the historically larger number of health professionals engaged in the clinical treatment of diseases, the ICHI classification also shows a strong clinical focus. In general, it was found that the ICHI criteria primarily involved functions (such as body regions or involved healthcare institutions) throughout general PH interventions which are often targeting diseases and specific diagnoses. Regarding the beta status of ICHI, the specifics of PH interventions might be integrated into future ICHI versions to support coordinated research and decisions in HTA worldwide.

## Conclusions

Over the last decades, considerable efforts have been deployed to act against the increasing burden of PH concerns overall. There is now enough hindsight to realize that many of the implemented strategies and interventions do not allow to put an end to the relentless spread of the so-called civilization diseases and recent outbreaks of vaccine-preventable diseases in a decisive manner.

HTA is a young discipline that originated from the need to support decision making on value in healthcare and, for the moment, the original disease-centered HTA focus still holds a dominating place, often at the expense of a more general health maintenance priority setting on the long term. This survey underlines a certain inadequacy of HTA from an overall PH perspective. Reshaping the current public health HTA methods and related practices of policy priority setting will be crucial for implementing efficient and effective interventions. The quickly expanding use of the HTA concepts by professionals and stakeholders from different horizons holds great promise for ameliorations in PH-oriented research and strategies across the different scientific and policy territories in spite of the challenges this poses (13, 49–51).

## Data Availability Statement

The datasets generated for this study are available on request to the corresponding author.

## Ethics Statement

Ethical approval for this study was not required in accordance with national legislation and institutional requirements due to fact that the data presented are anonymous, present no potential for identification of specific individuals with the dissemination of the research results, and are not considered to be sensitive or confidential in nature.

## Author Contributions

JS, IG-I, and IL-W contributed to the conception and design of the study. IG-I and ER organized the distribution of the survey. JS and ER extracted the survey data and performed the statistical analysis. JS, ER, and IL-W performed the initial classification of PH interventions. MW and SG performed the coding of PH interventions according to ICHI and wrote the sections of the manuscript. JS and IL-W wrote the first draft of the manuscript. All the authors contributed to manuscript revision as well as read and approved the submitted version.

## Conflict of Interest

The authors declare that the research was conducted in the absence of any commercial or financial relationships that could be construed as a potential conflict of interest.
