# Supplementary material for: Evaluating Public Health Interventions: A Neglected Area in Health Technology Assessment
Source: Front Public Health. 2020 Apr 22;8:106. doi: 10.3389/fpubh.2020.00106 (PMC7188782; doi:10.3389/fpubh.2020.00106)
Supplement: Supplementary file 1 [file Table_1.docx]

Supplementary Material

**Supplementary Table 1:** INPHORM Survey

*The aim of this survey is to identify which public health technologies are assessed by organizations working on health technology assessment (HTA) as well as what are the methodologies used, analyses performed and barriers to assessment process and decision implementation.*

*This survey is about HTA of health technologies in public health:*

- *Public Health refers to all organized measures (whether public or private) to prevent disease, promote health, and prolong life among the population as a whole, involving a collaborative effort by all parts of the health sector, working to ensure the well-being of society through comprehensive prevention, treatment, care and support.*
- *A health technology is defined as an intervention developed to prevent, diagnose or treat medical conditions; promote health; provide rehabilitation; or organize healthcare delivery (according to* [*http://htaglossary.net/health+technology*](http://htaglossary.net/health+technology)*). By definition this includes public health programs and services.*
- *Drugs are excluded from this survey, the focus is specifically on health technologies that allow to shift from “cost-cutting” and “disease management” to “****cost-avoiding****” and “****health management****” (e.g. vaccination programs, screening initiatives, nutrition and other interventions related to lifestyle, home care & coaching programs, educational health devices, etc.)*

**A. Contact Information**

1. Name (first, last):

_________________________

1. Name of organization:

*_________________________*

1. Country:

*_________________________*

1. E-Mail:

*_________________________*

**B. Activities related to evaluation of public health technologies**

1. Does your organization engage in any aspect of HTA in the area of public health, including topic selection, assessment, decisions, and implementation?

- Yes
- No

1. If **yes**, please indicate in what activities your organization is involved (select all that apply):

- Horizon scanning
- Identification of topic/of target (sub)populations
- Selection of topic/of target (sub)populations
- Project prioritization
- Assessment (including reviewing/generating evidence of effectiveness or cost-effectiveness)
- Decisions
- Design of the intervention/educational campaign/any form of care support
- Implementation
- Dissemination and diffusion
- Coordination of activities
- Monitoring
- Other (please specify): ___________________

1. If the answer is assessment, please specify in which aspects of the public health technology assessment your organization is involved:

- Medical aspects
- Organizational impact
- Economic evaluations
- Ethical considerations
- Societal perspectives and consequences
- Legal consequences
- Other (please specify): ___________________

1. If the answer is decisions, please specify the aspects of the evaluation that may influence the decision about the adoption of a public health technology (e.g. budget impact, the burden on health care resources, the burden on other public (i.e. non-health care resources), in evolution of NCDs, health literacy of the population …):

- *_____________________________________*

**C. Public Health Intervention Candidates**

1. Which is the total number of health technologies that your organization has evaluated since 2013?

_________________

Please provide the requested information below for each of the public health technology candidates considered for evaluation by your organization that you are aware of *.

Information for the public health intervention candidate*:

1. Name of Public health intervention candidate:

- ________________

1. Type of outcome (if not obtained yet, please specify the research question)*:

- ________________

1. Reasons for conducting assessment or implementing/adopting the public health interventions candidate*:

- To identify or clarify a public health need
- To estimate the healthcare burden of a public health concern
- To assess the impact of change in healthcare practices (e.g. additional home care, adjunctive support to existing therapeutic approaches, innovative coaching of healthcare professionals/of patients or at-risk population…)
- To support recent options benefitting from new research findings (trials on vaccines, data on sensitivity/specificity of screening initiatives, meta-analyses …)
- To quantify the impact (positive or negative) on healthcare budget/resources
- To determine whether a proposed public health technology represents a better alternative to standard procedures used
- To evaluate the potential impact of a future intervention
- On request of a commercial company who finances the assessment (in case of IT innovations for instance)
- Other (please specify): ___________________

*it is repeated for a number of each indicated public health intervention candidate

**D. Decision on a Public Health Technology**

1. Please indicate the methods, frameworks, or tools used by your organization to assess a public health technology (select all that apply):

- Health Technology Assessment
- Health Impact Assessment
- EUnetHTA core model
- INTEGRATE-HTA model
- Health Technology Reassessment
- Multi-Criteria Decision Analysis
- Program Budgeting Marginal Analysis
- Guideline for Not Funding Health Technologies (GuNFT)
- Model for Sustainability in Health care by Allocating Resources Effectively (SHARE)
- Budget-impact analyses
- Based on one of the four existing guidance documents
- Other (please specify): ___________________

* *Health Technology Assessment Of Public Health Interventions: A Synthesis Of Methodological Guidance. International Journal Of Technology Assessment In Health Care, 33:2 (2017), 135–146*

1. Please indicate the barriers experienced (or expected) by your organization in reaching a recommendation/decision about the implementation/adoption or delisting of a public health technology (select all that apply):

- Common methodological issues*** and lack of clear methodological frameworks to properly assess public health interventions through an HTA approach
- Lack of systematic decision process for Public Health management in the general population
- Lack of expertise to assess a public health technology that can only be evaluated on the long term (requires to invest resources well before achieving the result)
- Lack of relevant data to conduct assessment
- Reluctance to invest if there are costs with existing technology and supporting capital infrastructure that have been incurred and are not recoverable for sure
- Uncertainty about the potential benefits in unaware/unmotivated target populations
- The influence of well-established interest and/or advocacy groups
- Conflicting priorities among diverse stakeholders
- Sensitivity of Public Health target population (e.g., children, overweight citizens, undiagnosed conditions in at-risk groups)
- Usually a cross-sectorial and complex intervention, with a clear difficulty to assess the impact and to reallocate resources across and between programs or sectors
- Political challenges
- None
- Other (please specify): ___________________

*** Common methodological issues: Quality of evidence is often limited; Concerns related to outcome measurement in the absence of clinical settings (e.g. free-living elderly), especially among heterogeneous populations; Anticipated uncertainties in future outcomes or not; Lack of clearly identified paying parties etc.

1. Please indicate the barriers experienced by your organization to implementing a recommendation/decision on a public health technology (select all that apply):

- Insufficient timelines to implement decisions
- Difficulty in communicating and getting the message through to the general population
- Lack of funding for implementation
- Lack of skills in change management
- Lack of staff & resources necessary to implement the intervention
- Clinician reluctance to change habits in their daily practice
- Lack of perceived benefit from patient and citizen perspective
- Lack of perceived benefit related to complex context of the technology/bundle of technologies
- Perception that management priority is costing money while return on investment is not warranted
- Lack of incentives
- None
- Not applicable (i.e., my organization is not involved in implementing decisions on Public Health interventions)
- Other (please specify): ___________________

1. Even without precedent cases up until now, is your organization currently involved or planning to launch an evaluation of a public health technology?

- Yes
- No

1. If yes, please specify the nature of the public health technology as well as reasons and timeline for conducting the evaluation:

- ________________________

1. With regard to your own activities in this area, is there any other aspect you would like to address:

- ________________________
