# Supplementary material for: Evaluating Public Health Interventions: A Neglected Area in Health Technology Assessment
Source: Front Public Health. 2020 Apr 22;8:106. doi: 10.3389/fpubh.2020.00106 (PMC7188782; doi:10.3389/fpubh.2020.00106)
Supplement: Supplementary file 2 [file Table_2.docx]

Supplementary Material

**Supplementary Table 2:**

List of barriers experienced across 37 organizations when reaching a recommendation/decision about the implementation/adoption or delisting of a PH technology (Multiple choice question).

| Categories | N | %* |
| --- | --- | --- |
| *Lack of relevant data to conduct assessment* | 20 | 54 |
| *Conflicting priorities among diverse stakeholders* | 16 | 43 |
| *Common methodological issues* and lack of clear methodological frameworks to properly assess public health interventions through an HTA approach* | 12 | 32 |
| *Usually a cross-sectorial and complex intervention, with a clear difficulty to assess the impact and to reallocate resources across and between programs or sectors* | 11 | 30 |
| *Political challenges* | 11 | 30 |
| *Uncertainty about the potential benefits in unaware/unmotivated target populations* | 9 | 24 |
| *The influence of well-established interest and/or advocacy groups* | 9 | 24 |
| *Lack of expertise to assess a public health technology that can only be evaluated on the long term (requires to invest resources well before achieving the result)* | 7 | 19 |
| *Lack of systematic decision process for Public Health management in the general population* | 6 | 16 |
| *Not applicable (i.e., my organization is not involved in making decisions on public health issues)* | 6 | 16 |
| *Sensitivity of Public Health target population (e.g., children, overweight citizens, undiagnosed conditions in at-risk groups)* | 5 | 14 |
| *Reluctance to invest if there are costs with existing technology and supporting capital infrastructure that have been incurred and are not recoverable for sure* | 4 | 11 |
| *None* | 2 | 5 |
| *Other* | 1 | 3 |
| Total number of answers | 119 |  |

*percentage of institutions that answered positively;
