# Supplementary material for: Evaluating Public Health Interventions: A Neglected Area in Health Technology Assessment
Source: Front Public Health. 2020 Apr 22;8:106. doi: 10.3389/fpubh.2020.00106 (PMC7188782; doi:10.3389/fpubh.2020.00106)
Supplement: Supplementary file 3 [file Table_3.docx]

Supplementary Material

**Supplementary Table 3:**

List of barriers experienced across 37 organizations when implementing a recommendation/decision on a PH technology (Multiple choice question).

| Categories | N | % |
| --- | --- | --- |
| *Not applicable (i.e., my organization is not involved in implementing decisions)* | 15 | 41 |
| *Clinician reluctance to change habits in their daily practice* | 9 | 24 |
| *Lack of funding for implementation* | 8 | 22 |
| *Lack of staff & resources necessary to implement the intervention* | 8 | 22 |
| *Perception that management priority is costing money while return on investment is not warranted* | 8 | 22 |
| *Lack of perceived benefit related to complex context of the technology/bundle of technologies* | 7 | 19 |
| *Lack of skills in change management* | 5 | 14 |
| *Lack of incentives* | 5 | 14 |
| *Insufficient timelines to implement decisions* | 4 | 11 |
| *Difficulty in communicating and getting the message through to the general population* | 4 | 11 |
| *Lack of perceived benefit from patient and citizen perspective* | 3 | 8 |
| *Other* | 3 | 8 |
| *None* | 2 | 5 |
| Total number of answers | 81 |  |

*percentage of institutions that answered positively;
