# Supplementary material for: Evaluating Public Health Interventions: A Neglected Area in Health Technology Assessment
Source: Front Public Health. 2020 Apr 22;8:106. doi: 10.3389/fpubh.2020.00106 (PMC7188782; doi:10.3389/fpubh.2020.00106)

Supplementary Material

1 Supplementary Figure 1

Supplementary figure 1. Descriptive analysis of 52 institutions responding to the survey (frequency of different types of organizations expressed as percentages)

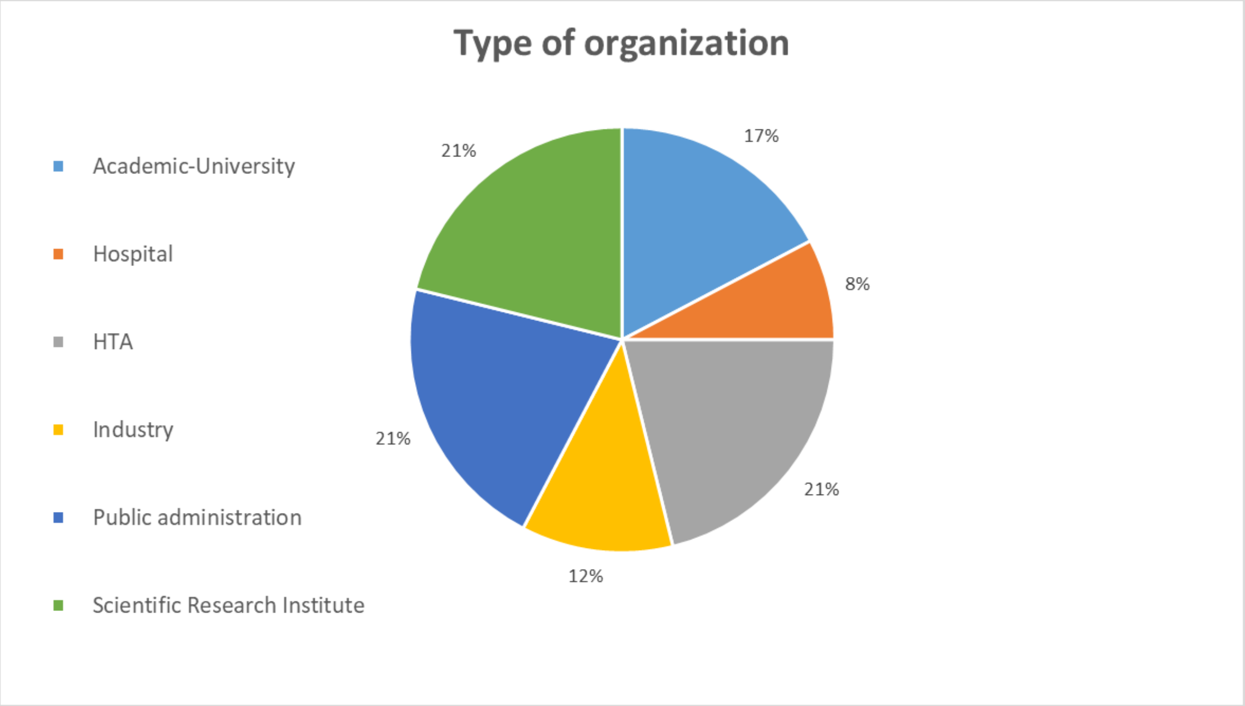

2 Supplementary Figure 2

Supplementary figure 2. Descriptive analysis of 52 institutions responding to the survey (frequency of different continents expressed as percentages)

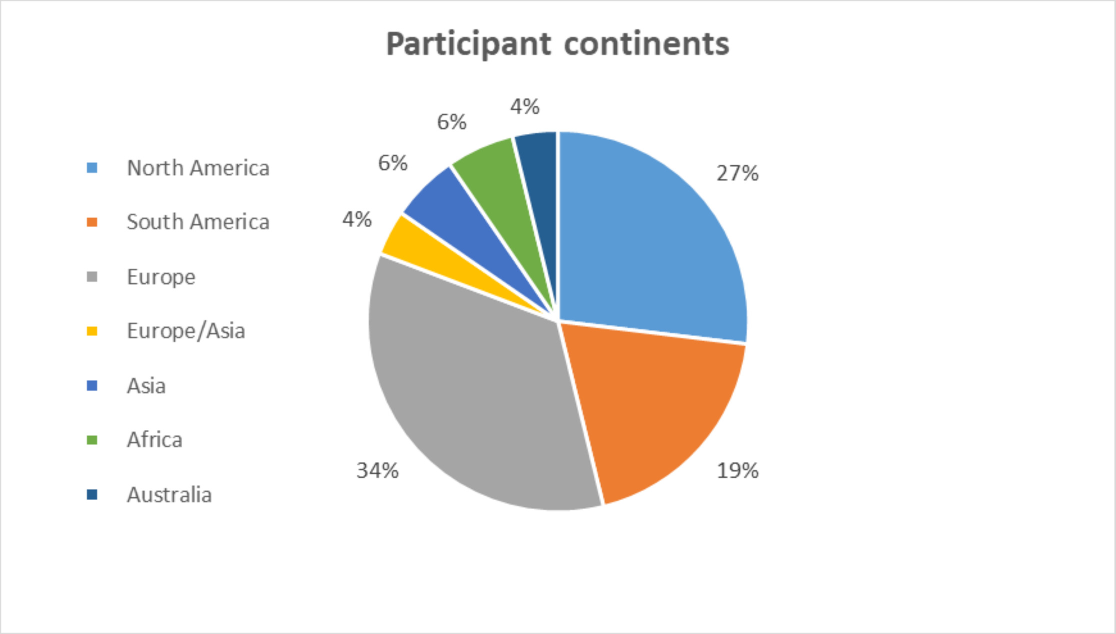

Supplement: Supplementary file 4 [file Image_1.pdf]
